# Supplementary material for: Acute ACAT1/SOAT1 Blockade Increases MAM Cholesterol and Strengthens ER-Mitochondria Connectivity
Source: Int J Mol Sci. 2023 Mar 14;24(6):5525. doi: 10.3390/ijms24065525 (PMC10059652; doi:10.3390/ijms24065525)
Supplement: Supplementary file 1 [file ijms-24-05525-s001.zip › ijms-2259506-supplementary.pdf]

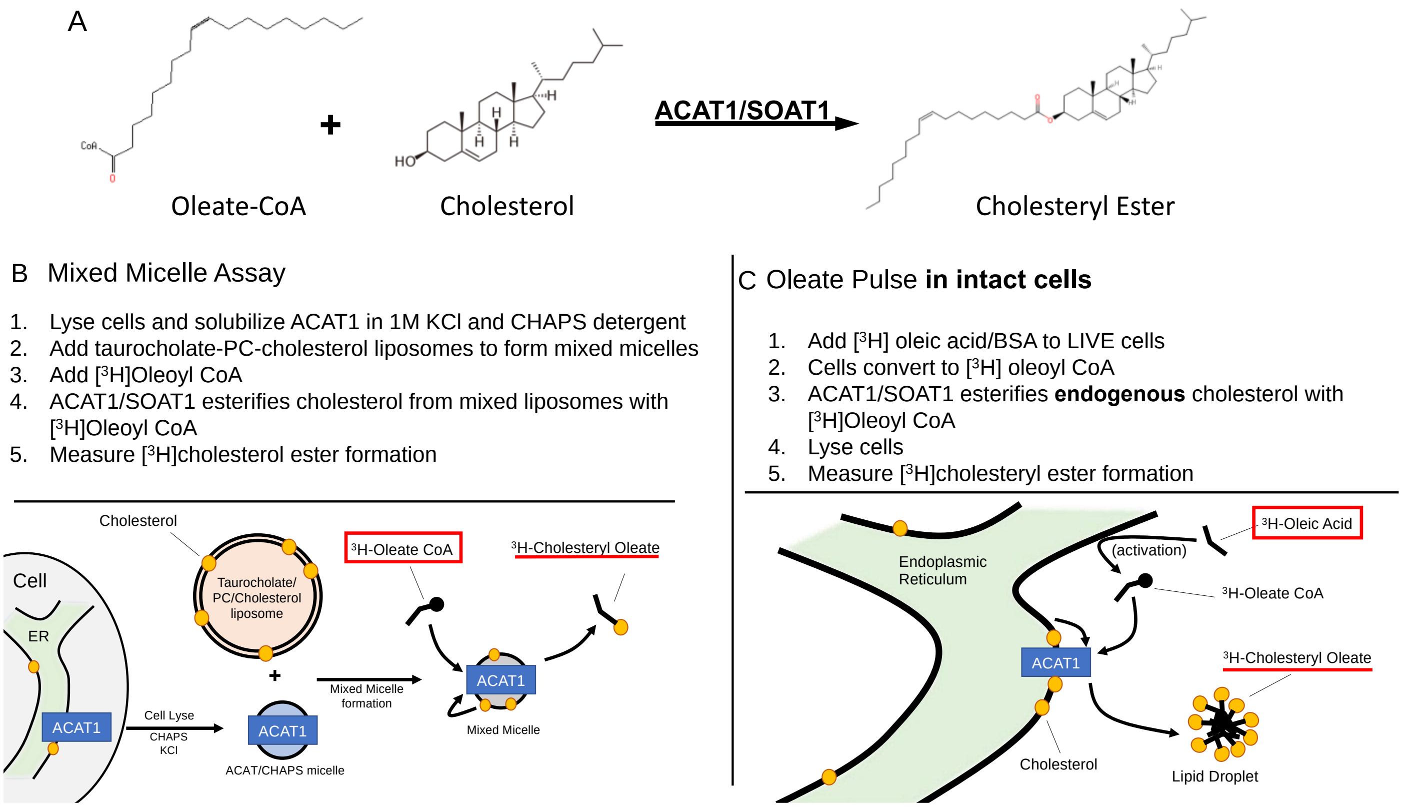

Figure S1 ACAT1/SOAT1 activity assays. A) Cholesterol esterification reaction catalyzed by ACAT1/SOAT1. B) Mixed micelle assay. ACAT1/SOAT1 from cells is solubilized in 1M KCl and CHAPS detergent to form ACAT1/CHAPS micelles and suspended in solution with taurocholate/phosphatidylcholine/cholesterol liposomes. Reconstituted ACAT1/SOAT1 esterifies excess micellar cholesterol with [^{3}H]oleate CoA. ACAT1/SOAT1 activity measured by [^{3}H] cholesteryl-oleate formation. C) [^{3}H] oleate pulse. [^{3}H] oleic acid added to live cells and activated with coenzyme A. ACAT1/SOAT1 activity measured by [^{3}H] cholesteryl-oleate formation. Glycerolipid synthesis measured by [^{3}H] oleic acid incorporation into other lipid species.

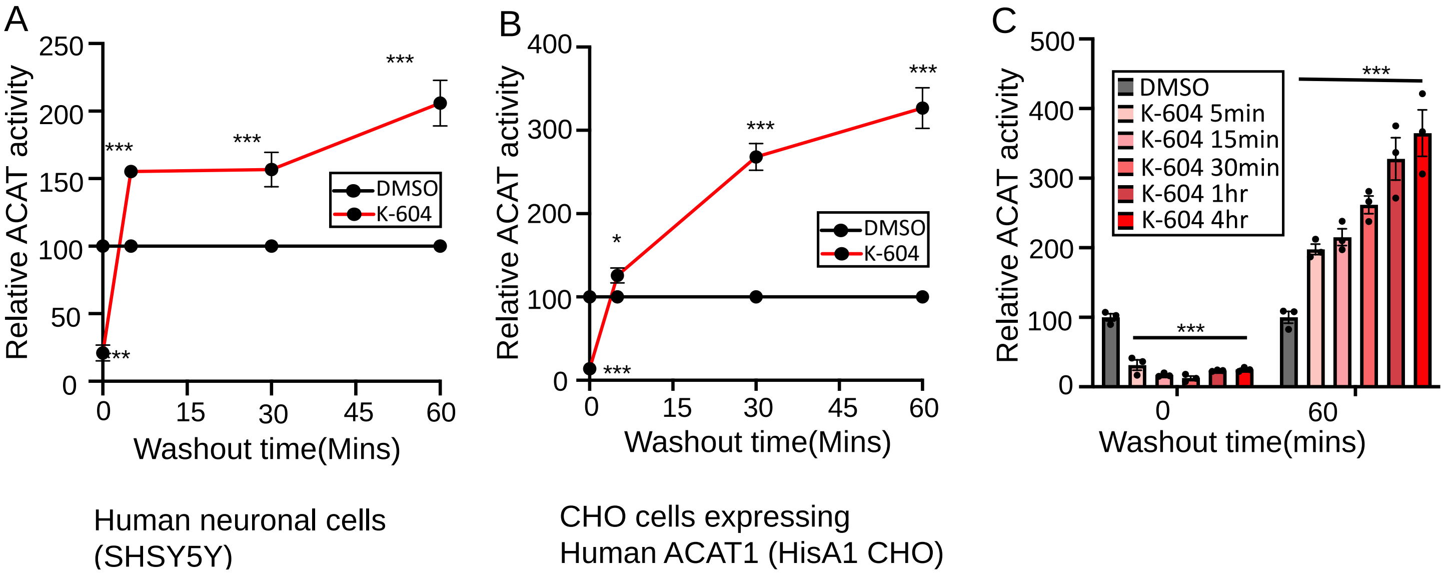

Figure S2 ACAT1/SOAT1 blockade cholesterol pool observation. A) ACAT1/SOAT1 activity as measured by $[^3H]$ Oleate pulse in SHSY5Y cells (immortalized human neuronal cell line). ACAT1 activity spike is seen in cells pre-treated with K-604 and washed with conditioned drug-free media. n= 6. B) ACAT1/SOAT1 activity as measured by $[^3H]$ oleate pulse in hACAT1 CHO cells (CHO cells expressing human ACAT1/SOAT1). ACAT1 activity spike is seen in cells pre-treated with K-604 and washed with conditioned drug-free media. n= 3. C) N9 cells pre-treated with K-604 for varying amounts of time. Cells were washed or not with conditioned drug-free media for 60 minutes before measuring ACAT1/SOAT1 activity with $[^3H]$ oleate pulse. n=3. Error bars represent SEM. p-value determined using student's t-test; *p<0.05; **p<0.01; ***p<0.001.

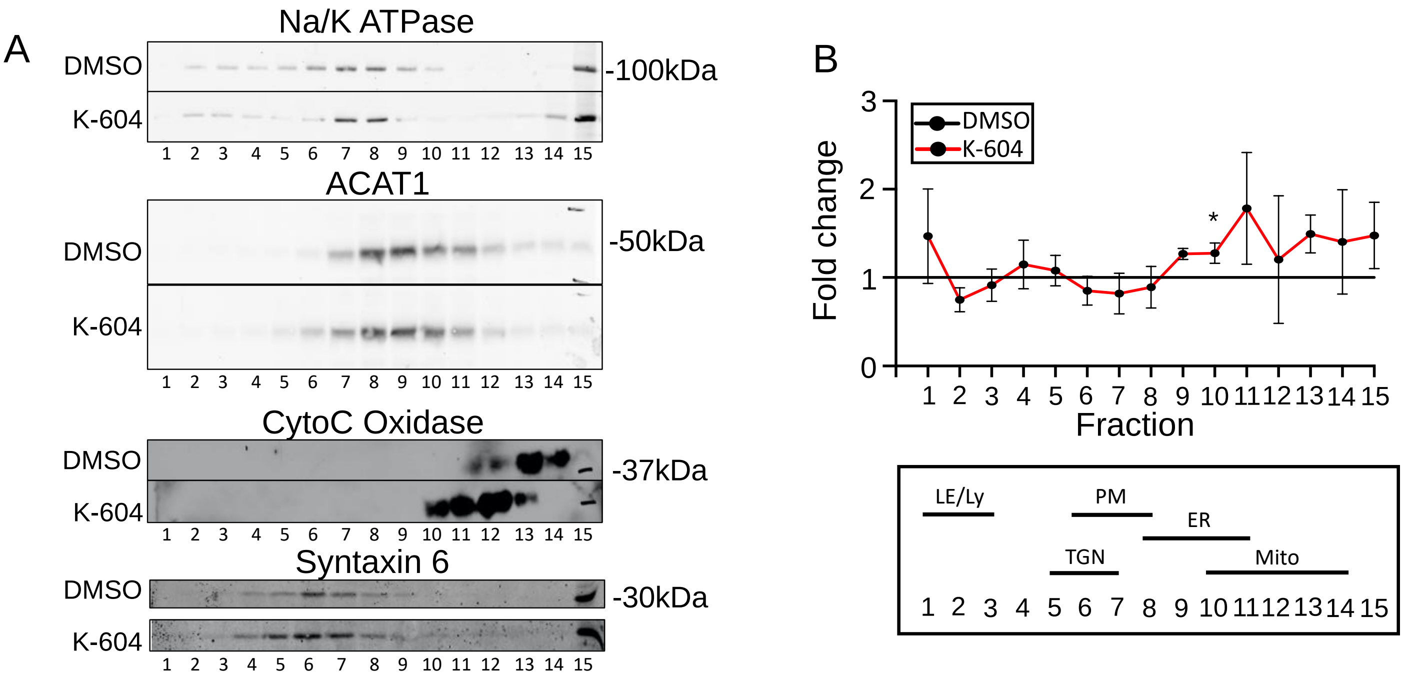

Figure S3 Measuring cholesterol in cellular fractions separated by OptiPrep fractionation. N9 cells were treated with 0.5µM K-604 or DMSO vehicle control for 4 hours before lysing cells and separating components with OptiPrep fractionation. A) representative Western blots showing distribution of plasma membrane (Na/K ATPase), endoplasmic reticulum (ACAT1/SOAT1), mitochondria (cytochrome C Oxidase), and trans golgi apparatus (Syntaxin 6). B) quantification of cholesterol analysis performed for OptiPrep fractions using thin layer chromatography. n=3. Error bars represent SEM. p-value determined using student's t-test; *$p<0.05$

Gene Name	logFC	P.Value	UniProt ID	Description	Gene Name	logFC	P.Value	UniProt ID	Description
Mtx3	0.1927	0.011	D3YTP3	Metaxin	Vapa	0.102083	0.4471	Q9WV55	Vesicle-associated membrane protein-associated protein A
Soat1	0.1954	0.018	Q61263	Sterol O-acyltransferase 1	Zdhhc5	0.053328	0.46369	Q8VDZ4	Palmitoyltransferase ZDHHC5
Exd2	0.1987	0.021	Q8VEG4	Exonuclease 3-5 domain-containing protein 2	Mief1	0.065607	0.47951	Q8BGV8	Mitochondrial dynamics protein MID51
Maco1	0.2272	0.029	Q7TQE6	Macoilin	Ubxn4	0.079242	0.49984	A0A0R4J07	UBX domain-containing protein 4
Nap1l4	-0.1886	0.032	B7ZNL2	Nap1l4 protein	Stx5a	0.059767	0.50269	H3BJ02	Syntaxin 5A
Elovl1	0.1873	0.034	Q9JLJ5	Elongation of very long chain fatty acids protein 1	Pkd2	0.061474	0.5203	O35245	Polycystin-2
Bet1	-0.2925	0.045	O35623	BET1 homolog	Ptrh2	0.051374	0.53003	Q8R2Y8	Peptidyl-tRNA hydrolase 2, mitochondrial
Dnajc14	0.1596	0.049	Q921R4	DnaJ homolog subfamily C member 14	Chp1	0.042547	0.53279	P61022	Calcineurin B homologous protein 1
Tmpo	-0.3428	0.051	Q61029	Lamina-associated polypeptide 2, isoforms beta/delta/epsilon/gamm	Slc27a4	0.041058	0.53443	Q91VE0	Long-chain fatty acid transport protein 4
Bax	-0.2027	0.057	A0A1B0GT	Apoptosis regulator BAX	Camlg	-0.06527	0.53497	P49070	Guided entry of tail-anchored proteins factor CAMLG
Sar1a	-0.2174	0.063	Q99JZ4	GTP-binding protein SAR1a	Itgb1	-0.05067	0.55277	P09055	Integrin beta-1
Ykt6	-0.1828	0.064	Q9CQW1	Synaptobrevin homolog YKT6	Vezt	0.047093	0.56229	D3Z4E6	Vezatin
Cdk5rap3	0.2752	0.068	Q99LM2	CDK5 regulatory subunit-associated protein 3	Rhot2	0.05142	0.56758	Q8JZN7	Mitochondrial Rho GTPase 2
Armcx3	0.1394	0.07	Q8BHS6	Armadillo repeat-containing X-linked protein 3	Lrrc59	-0.04798	0.5681	Q922Q8	Leucine-rich repeat-containing protein 59
Gdap1l1	-0.1341	0.078	A2A5H8	Ganglioside-induced differentiation-associated protein 1-like 1	Tmem9b	0.041893	0.56911	Q9JJR8	Transmembrane protein 9B
Mrps7	0.2085	0.095	Q80X85	28S ribosomal protein S7, mitochondrial	Sec61b	-0.05387	0.57486	Q9CQS8	Protein transport protein Sec61 subunit beta
Ckap4	-0.1534	0.101	Q8BMK4	Cytoskeleton-associated protein 4	Dhcr7	0.07808	0.57678	A0A140LIT	7-dehydrocholesterol reductase
C1qbp	0.1967	0.108	Q8R5L1	Complement component 1 Q subcomponent-binding protein	Srprb	0.040228	0.59727	P47758	Signal recognition particle receptor subunit beta
Lemd3	-0.1456	0.115	E9QP59	Inner nuclear membrane protein Man1	Pdia6	-0.06395	0.62205	Q922R8	Protein disulfide-isomerase A6
Myo19	0.143	0.117	Q5SV80	Unconventional myosin-XIX	Ddrgk1	0.05077	0.62891	Q80WW9	DDRGK domain-containing protein 1
Mtfr1l	0.1528	0.126	Q9CWE0	Mitochondrial fission regulator 1-like	Pgrmc1	0.048194	0.63992	O55022	Membrane-associated progesterone receptor component 1
Ephx1	-0.1512	0.142	Q9D379	Epoxide hydrolase 1	Mavs	-0.06122	0.64171	Q8VCF0	Mitochondrial antiviral-signaling protein
Fkbp8	0.156	0.146	O35465	Peptidyl-prolyl cis-trans isomerase FKBP8	Erlin2	-0.03081	0.66071	Q8BFZ9	Erlin-2
Spcs2	-0.1105	0.158	A0A140LJ0	Signal peptidase complex subunit 2 (Fragment)	Acbd5	0.031634	0.67527	E9QNH7	Acyl-CoA-binding domain-containing protein 5
Jund	-0.3013	0.159	P15066	Transcription factor JunD	Emd	0.037047	0.69371	I7HJS1	Emerin
Sc5d	0.1894	0.162	O88822	Lathosterol oxidase	Ociad1	0.027402	0.69749	Q9CRD0	OCIA domain-containing protein 1
Lbr	-0.2153	0.168	Q3U9G9	Delta(14)-sterol reductase LBR	Vkorc1l1	0.038791	0.70187	Q6TEK5	Vitamin K epoxide reductase complex subunit 1-like protein 1
Sar1b	-0.1287	0.177	Q9CQC9	GTP-binding protein SAR1b	Cisd2	-0.02589	0.70347	Q9CQB5	CDGSH iron-sulfur domain-containing protein 2
Pcca	0.1669	0.186	Q91ZA3	Propionyl-CoA carboxylase alpha chain, mitochondrial	Emc7	-0.02619	0.707	Q9EP72	ER membrane protein complex subunit 7
Alg9	-0.0933	0.195	Q8VDI9	Alpha-1,2-mannosyltransferase ALG9	Erlin1	-0.02532	0.7291	Q91X78	Erlin-1
Rft1	0.11	0.207	Q8C3B8	Protein RFT1 homolog	Vma21	-0.02658	0.73464	Q78T54	Vacuolar ATPase assembly integral membrane protein Vma21
Cyb5r3	-0.0948	0.216	Q9DCN2	NADH-cytochrome b5 reductase 3	Mlf2	-0.02245	0.7518	Q99KX1	Myeloid leukemia factor 2
Rab29	0.1148	0.216	Q91YQ1	Ras-related protein Rab-7L1	Tex264	-0.02589	0.76034	E9Q137	Testis-expressed protein 264 homolog
Dnajc11	0.1126	0.221	Q5U458	DnaJ homolog subfamily C member 11	Atp6ap2	0.024313	0.76287	Q9CYN9	Renin receptor
Praf2	-0.0986	0.224	Q9JIG8	PRA1 family protein 2	Smim12	0.027544	0.76714	Q78RX3	Small integral membrane protein 12
Pigu	0.1113	0.225	Q3TAA8	Phosphatidylinositol glycan anchor biosynthesis class U protein	Faf2	-0.02022	0.7674	Q3TDN2	FAS-associated factor 2
Agpat1	0.1009	0.227	A0A0R4J26	1-acyl-sn-glycerol-3-phosphate acyltransferase	Mmgt1	0.022617	0.78859	Q8K273	ER membrane protein complex subunit 5
Atp2a2	0.1085	0.232	O55143	Sarcoplasmic/endoplasmic reticulum calcium ATPase 2	Dhrs7b	0.016804	0.79354	Z4YKT6	Dehydrogenase/reductase SDR family member 7B
Fundc2	0.085	0.264	Q9D6K8	FUN14 domain-containing protein 2	Sec63	0.01683	0.81793	Q8VHE0	Translocation protein SEC63 homolog
Hmox2	-0.0789	0.265	O70252	Heme oxygenase 2	Stt3b	-0.01708	0.83568	A0A0R4J0D	Dolichyl-diphosphooligosaccharide--protein glycotransferase
Pex14	-0.0855	0.267	Q9R0A0	Peroxisomal membrane protein PEX14	Vapb	-0.0244	0.83767	Q8BH80	Vesicle-associated membrane protein, associated protein B and C
Ube2j1	0.1358	0.276	Q9JJZ4	Ubiquitin-conjugating enzyme E2 J1	Aldh3a2	0.01015	0.88328	B1AV77	Aldehyde dehydrogenase
Ubxn8	-0.2942	0.276	Q3TTF2	UBX domain-containing protein 8	Rab1A	0.01746	0.88415	P62821	Ras-related protein Rab-1A
Bcap31	-0.0923	0.278	Q61335	B-cell receptor-associated protein 31	Tmem214	0.009196	0.90629	D3Z6S1	Transmembrane protein 214
Tmem209	-0.0997	0.295	Q8BRG8	Transmembrane protein 209	Akap13	0.011075	0.90662	E9Q394	A-kinase anchor protein 13
Tmx1	-0.0819	0.304	Q8VBT0	Thioredoxin-related transmembrane protein 1	Lnpk	-0.0086	0.90883	Q7TQ95	Endoplasmic reticulum junction formation protein lunapark
Pgrmc2	-0.0736	0.326	Q80UU9	Membrane-associated progesterone receptor component 2	Tmem230	-0.01252	0.91239	Q8CIB6	Transmembrane protein 230
Tdrkh	0.113	0.344	A0A0G2JE	Tudor and KH domain-containing protein	Gpat4	-0.00918	0.91849	Q8K2C8	Glycerol-3-phosphate acyltransferase 4
Reep5	-0.074	0.349	G3X8R0	Receptor expression-enhancing protein	Cisd1	-0.00836	0.92439	Q91WS0	CDGSH iron-sulfur domain-containing protein 1
Ormdl2	0.0731	0.353	Q9CQZ0	ORM1-like protein 2	Emc6	0.009607	0.93976	Q9CQW0	ER membrane protein complex subunit 6
Atg2a	0.2378	0.388	F6V3Y9	Autophagy-related protein 2 homolog A (Fragment)	Tmem201	0.007202	0.94059	A2A8U2	Transmembrane protein 201
Tomm5	0.0756	0.396	B1AXP6	Mitochondrial import receptor subunit TOM5 homolog	Abcd3	-0.00503	0.94599	P55096	ATP-binding cassette sub-family D member 3
Rabl3	0.0569	0.4	Q9D4V7	Rab-like protein 3	Usp30	-0.00526	0.94789	Q3UN04	Ubiquitin carboxyl-terminal hydrolase 30
Tmem199	-0.0741	0.403	Q5SYH2	Transmembrane protein 199	Tomm20	0.005444	0.94907	Q9DCC8	Mitochondrial import receptor subunit TOM20 homolog
Ssr1	-0.0555	0.405	Q9CY50	Translocon-associated protein subunit alpha	Use1	-0.00556	0.95185	Q9CQ56	Vesicle transport protein USE1
Scd2	0.1247	0.407	A0A494BA	Stearoyl-CoA desaturase 2	Dgke	-0.00335	0.96596	Q9R1C6	Diacylglycerol kinase epsilon
Rap2c	0.0634	0.411	Q8BU31	Ras-related protein Rap-2c	C2cd2l	-0.0024	0.97951	Q80X80	Phospholipid transfer protein C2CD2L
Tbl2	0.0568	0.413	Q9R099	Transducin beta-like protein 2	Sec22b	0.001428	0.98666	O08547	Vesicle-trafficking protein SEC22b
Lpgat1	0.06	0.421	E9QL80	Acyl-CoA:lysophosphatidylglycerol acyltransferase 1	Dnm1l	0.000872	0.99281	E9PUD2	Dynamin-1-like protein
Tor1aip1	-0.0584	0.437	E9PWW2	Torsin-1A-interacting protein 1	Rnf5	-0.00079	0.99327	O35445	E3 ubiquitin-protein ligase RNF5
Tmem109	-0.0487	0.446	D3Z0I8	Transmembrane protein 109 (Fragment)	Abca3	-0.00047	0.9954	Q8R420	Phospholipid-transporting ATPase ABCA3

Figure S4 MAM proteins identified in proteomic analysis. N9 cells were treated with 0.5µM K-604 or DMSO vehicle for 4 hours before subject to MAM fractionation. MAM fractions were analyzed by protein mass spec. this table displays the fold change (K-604 vs vehicle) and p-values of MAM proteins identified in Kwak et al. [108]
